# Community composition and seasonal dynamics of microplastic biota in the Eastern Mediterranean Sea

**DOI:** 10.1038/s41598-024-73281-3

**Published:** 2024-10-30

**Authors:** Keren Davidov, Katherine S. Marsay, Sheli Itzahri, Maxim Rubin-Blum, Paula Sobral, Chana F. Kranzler, Matan Oren

**Affiliations:** 1 Department of Molecular Biology, Ariel University, Ariel, Israel; 2 Israel Oceanographic and Limnological Research, National Institute of Oceanography, Tel Shikmona, Haifa, Israel; 3 MARE - Marine and Environmental Sciences Centre & ARNET - Aquatic Research Network Associated Laboratory, NOVA School of Science and Technology, NOVA University of Lisbon, Lisbon, Portugal; 4 Mina & Everard Goodman Faculty of Life Sciences, Bar-Ilan University, Ramat Gan, Israel

**Keywords:** Plastisphere, Microplastic, Diatoms, Eastern Mediterranean Sea, Biodiversity, Seasonality, Microbiome, Nanopore, 16S, 18S, Metabarcoding, Biodiversity, Ecology, Microbiology, Ecology, Environmental sciences, Ocean sciences

## Abstract

Marine plastic pollution poses a growing environmental threat, with microplastics accumulating in the global oceans. This study profiles the seasonal dynamics and taxonomic composition of the plastisphere, the microplastic ecosystem, in the Eastern Mediterranean Sea. Using long-read 16 S and 18 S metabarcoding, we analyzed offshore microplastic and whole seawater samples across each season over a two-year period. The analysis revealed a higher richness of prokaryotic communities on microplastics compared to seawater, which was predominantly composed of Proteobacteria and Bacteroidota and exhibited notable seasonal variability. Benthic eukaryotes were enriched on microplastics compared to the surrounding seawater. Diatoms (Bacillariophyceae), in particular, showed significant enrichment within the microplastic eukaryotic community with primarily pennate diatoms of *Amphora*, *Navicula*, and *Nitzschia* genera, whereas the seawater included mostly centric diatoms. Seasonal fluctuations were less pronounced in the microplastic communities than in seawater, highlighting the relative stability of this new human-made ecosystem. These findings underscore the unique ecological niche of microplastic-associated communities in marine environments.

**Supplementary Information:**

The online version contains supplementary material available at 10.1038/s41598-024-73281-3.

## Introduction

Marine plastic pollution is an increasing environmental concern as the quantities of plastic entering our oceans rise^1,2^. Over time, due to heat, light and UV radiation, sheering forces of currents and waves and biogenic activity, plastic items break down into smaller microplastic and nano-plastic particles. Generally defined as particles in the 1 μm to 5 mm size range^3^. Microplastics have been found in marine environments worldwide^4,5^, with high concentrations in large mid-ocean gyres^6^. Due to plastic durability, micro- and nano-plastic debris accumulate and persist in the marine environment at the sea surface, throughout the water column, and at the seafloor and beaches, posing significant risks to marine life^7^. Microplastics also serve as stable substrates for the colonization of marine organisms. It was repeatedly demonstrated that the biological composition of marine plastic debris differs from that of the surrounding seawater^8^. Therefore, plastic surfaces and their associated biota are considered a distinct ecosystem called the “plastisphere”^9^. The plastisphere ecosystem hosts a complex community of primary producers, predators and symbionts from different taxonomic groups, including bacteria and archaea, single-celled eukaryotes, fungi, algae and invertebrates^10^. The plastisphere may also contain potential pathogens (e.g. Refs.^11,12^). as well as hydrocarbon-degrading taxa (e.g. Refs.^13,14^).

The plastisphere community is affected by versatile environmental variables such as temperature, salinity, nutrients, dissolved oxygen and CO_2_, light, pH, water turbulence, and microplastic properties such as polymer type and color^8,15–17^. The two factors that have the strongest effect on these variables are the geographic location and the time of the year (i.e. the season). Indeed, the spatiotemporal characteristics of microplastic samples are considered central in shaping the plastisphere community composition^18,19^. Several studies investigated the seasonal variations in the plastisphere microbiome either experimentally^20–22^ or by analyzing microplastic samples collected from the marine environment^12,23^. However, the existing seasonal data is incomplete and lacks comprehensive analysis of both prokaryotic and eukaryotic taxa for each season across multiple years. Notably, seasonal data from the Mediterranean Sea is missing.

The Mediterranean Sea is a known sink for microplastics as it is an enclosed basin surrounded by highly populated coasts^24^. Microplastic concentrations as high as 324 particles/m^3^ (mean = 7.68 ± 2.38 particles/m^3^) were recorded in the Eastern Mediterranean Sea (EMS) waters along the Israeli coastline^25^. This study examined the composition and seasonal dynamics of marine microplastic debris and their attached biota near the Israeli Mediterranean coast. It provides insights into the prokaryotic and eukaryotic plastisphere and planktonic communities in the EMS in each season throughout the years 2020–2021.

## Methods

### Sampling

**Fig. 1:**
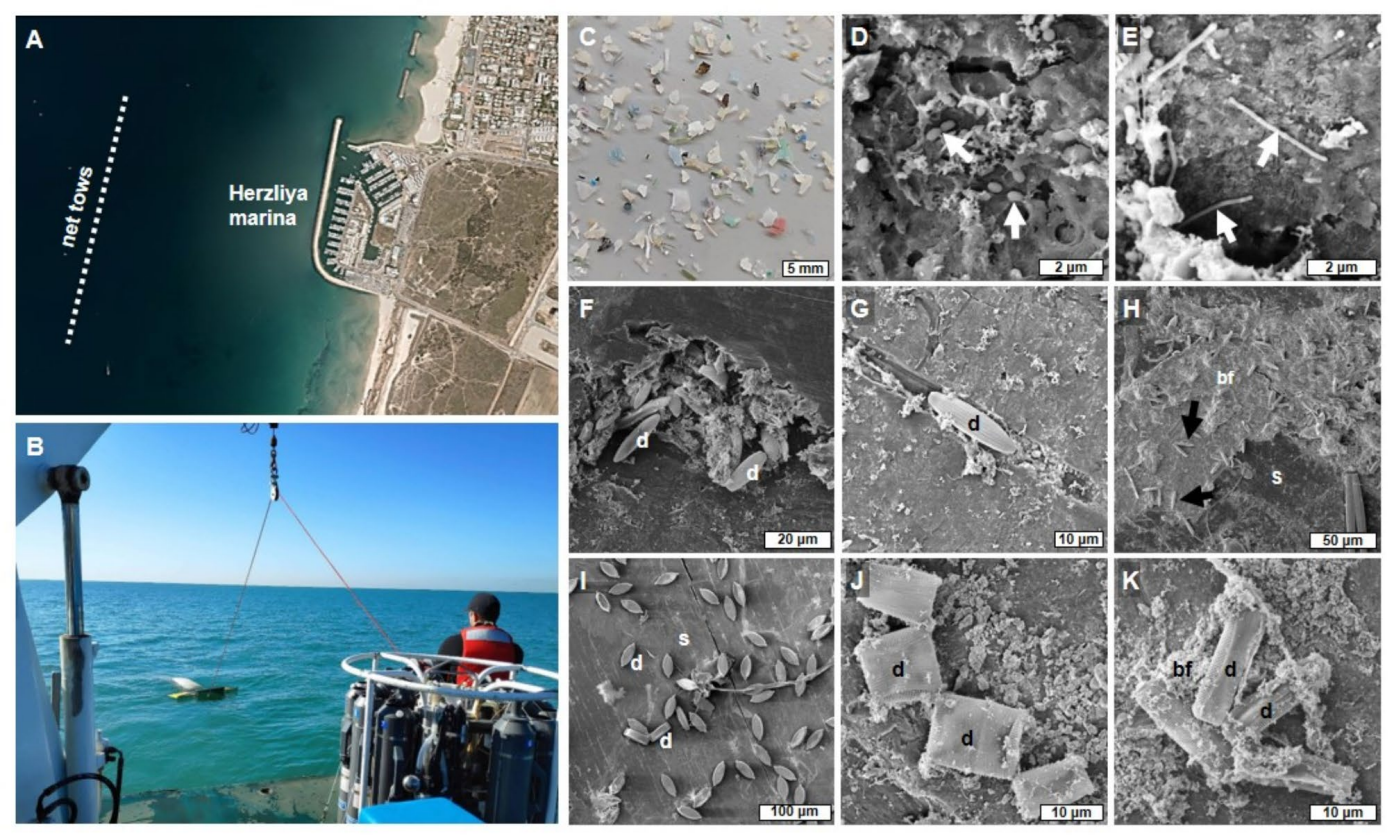
Bacteria and diatoms on sea-surface microplastic. (**A**) Sampling location 1–1.2 km offshore Herzliya, Israel. The dashed lines indicate the course of net tows. The satellite image was created using Google Maps (https://www.google.com/maps). (**B**) Manta net used to collect > 300 μm water-surface microplastic debris. This picture was taken by Matan Oren. (**C**) microplastic debris after manual separation. (**D**) Cocci-shaped bacteria in microplastic surface pits (white arrows). (**E**) Rod-shaped bacteria (white arrows). (**F**) Diatoms partially covered with biofilm inside a crack in the surface. (**G**) A pennate diatom is attached to a scratch on the surface. (**H**) Pennate diatoms (black arrows) on top of a biofilm layer. (**I**) Pennate diatoms directly attached to the surface. (**J**) Dividing diatoms on the microplastic surface. (**K**) Diatoms partially covered with biofilm. d-diatom/s, bf-biofilm, s-plastic surface.

Microplastic and seawater samples were collected from the water surface layer, 1–1.2 km from the shoreline of Herzliya, Israel (32°06’10.1"N, 34°46’51.2"E, Fig. 1A). The samples were collected using a manta net (Hydro-Bios, Microplastic net, 438217) with a mesh hole size of ~ 300 μm (Fig. 1B) in every season, as detailed in Table 1. The net was deployed from a research vessel (Mediterranean Explorer, https://www.ecoocean.org/en/) and towed parallel to the coastline at 2–3 knots for 20–30 min, with three repetitions along the same transect. Particles recovered from the collection cup (at the tip of the net) were rinsed with autoclaved filtered (0.22 μm) artificial seawater (40 ppt, Red Sea salt, Red Sea Inc.). Additionally, seawater from the endpoint of the net towing was sampled using sterile bottles. For DNA metabarcoding analysis of the plastic microbiome, a minimum of ten microplastic particles were randomly selected from each sample. To analyze the water microbiome, 0.5 L of whole seawater was filtered through a 0.22 μm polyethersulfone membrane (Millipore) using a 20 L/min pump (MRC). To characterize the microplastic composition of colors and shapes, the samples were filtered using metal sieves to collect debris between 0.3 and 5 mm in diameter, separated using fine tweezers and visualized under a magnifying stereoscope. Environmental parameters of the water column were measured using a CTD device attached to a rosette sampler at a depth of 0.5–1.5 m.

**Table 1:** Environmental parameters in each sample collection*.

	Winter 2020	Spring 2020	Summer 2020	Autumn 2020	Winter 2021	Spring 2021	Summer 2021	Autumn 2021
Sampling date	26.01	27.04	19.07	13.10	3.02	18.04	13.07	24.10
Day length (hr:min)	10.34	13.2	13.58	11.27	10.43	13.03	13.58	11.07
Sea surface temperature (°C)	17	20.8	29.4	28.5	18.7	19.9	29.8	26.2
Salinity (PSU)	38.5	38.8	39.1	39.4	39	39	39.4	39.5
Turbidity (FTU)	2.5	0.4	0.8	0.6	2.7	0.9	1.1	0.4
Fluorescence mg Chl/m^3^	0.7	0.34	0.86	0.51	0.22	0.35	0.17	0.14
PAR** (uE/m^2^/sec)	666	404	279	452	200	677	112	–
SbeopoxPS (%)***	102.18	100.94	100.32	100.44	98.26	103.11	100.38	91.21

*Parameters values represent the average of measurements taken by a CTD at depths of 0.5 to 1.5 m at the time of microplastic collection. **Photosynthetically active radiation. ***Oxygen saturation.

### SEM imaging

Scanning electron microscopy (SEM) was employed to visualize microorganisms on the polyethylene (PE) surface. PE samples underwent fixation for 2–5 h in a solution containing 1% glutaraldehyde and 4% paraformaldehyde (PFA) for one hour at room temperature. Subsequently, samples were washed three times for 5 min each in distilled water and stored in 50% ethanol in phosphate-buffered saline (PBS) at − 20 °C until further use. Before imaging, samples were dehydrated using a graded ethanol series (50%, 70%, 85%, 95%, and 100% ethanol) for 10 min each, followed by three additional 15-minute incubations in 100% ethanol. Dehydrated samples were then air-dried for a minimum of 5 h in a hood, sputter-coated with a 10 nm layer of platinum/gold using a Quorum Q150T ES instrument, and subsequently imaged using an Ultra-High-Resolution Maia 3 field-emission scanning electron microscope (FE-SEM) (Tescan) at a voltage range of 3–7 Kv.

### PCR and MinION sequencing

DNA was extracted from the microplastic particles of each sample using the DNeasy PowerWater Kit (Qiagen). The following amplification reactions (PCR) were performed using 25–75 ng of template DNA. The PCR products were cleaned with a QIAquick-PCR Purification kit (QIAGEN) to meet the criteria of the MinION nanopore library preparation protocol^26^. The near-complete 16S rRNA gene (16S in short) was amplified and sequenced using Oxford Nanopore 16S barcoding kit 24 (SQK-16S024) according to manufacturer instructions, generating ~ 1.5 kb-long amplicon sequences. For the barcoding of eukaryotes, the V4 - V5 region of the 18S rRNA gene (18S in short) was amplified with 566F (5’ - CAGCAGCCGCGGTAATTCC − 3’) and 1289R (5’ -ACTAAGAACGGCCATGCACC − 3’) primers to generate ~ 0.7 kb long sequences according to Ref.^14^. The 18 S sequencing libraries were prepared using the Native barcoding (EXP-NBD104) and ligation (SQK-LSK109) kits and protocols. The 16 S and 18 S multiplexed libraries were loaded onto separate MinION Nanopore Spot-on flow cells (FLO- MIN106D, version R9) and sequenced for 72 h or until reaching ~ 7 Giga nucleotides (~ 4 M reads). Base-calling for all libraries was carried out by the Guppy base calling software 3.3.3, using the MinKnow program with the “high accuracy” option. Raw reads were obtained in FAST5 and FASTq formats from which “pass” quality reads were subjected to further analysis.

### Sequence analysis and bioinformatics

The amplification, sequencing, and bioinformatics procedures followed those outlined by Ref.^14^. Subsequent processing and analysis of the generated reads utilized the MetONTIIME pipeline and QIIME2 plugins^27^. Reads were demultiplexed, and adaptors and PCR primers were trimmed. Then, sequences underwent quality filtering (min_quality 10) and length filtering. The length filtering was determined by read length histograms, with amplicon lengths falling within specific ranges (amplicon_length X, lenfil_tol Y): for 16S sequences, 1300–1600 nucleotides, and 650–1000 nucleotides for 18S sequences. Clustering and taxonomic classification were performed separately for each barcode. Sequences were clustered into consensus sequences with default MetONTIIME pipeline parameters (de novo strategy, clustering threshold parameter perc-identity 1). Taxonomy was assigned to the 16S and 18S amplicons using BLAST against the Silva132 database^28^, with a 90% identity threshold. All downstream analyses were based on relative abundance values, calculated by dividing the number of reads for each taxon in a sample by the total reads count. The analyses of alpha diversity, beta diversity (PCA, and abundance bar charts) were created by MicrobiomeAnalyst 2.0 web platform^29^. Datasets of individual samples (16S and 18S) under 10k reads were removed, while all other datasets were verified to 10k to enable reliable comparisons. We used the Masllin2 package in R to observe significant variation across seasons to perform a linear mixed-effects model analysis, associating microbial abundances with the season^30^. This analysis was based on OUT- level relative abundances with ‘Season’ as a fixed effect, and ‘Temperature (C)’, ‘Salinity (PSU)’, and ‘Year’ as random effects, using ‘Summer’ as the reference level. The same parameters were applied to the ‘water’ sample data. Common features between the ‘plastic’ and ‘water’ datasets were identified, filtered, and merged into a single data frame. The results were then filtered to retain only those with a q-value (adjusted p-value) less than 0.01.

## Results

### Environment parameters and microplastic characteristics

At each sampling timepoint during the two-year duration of our study, we measured several key environmental parameters, including sea surface temperature, salinity, turbidity, fluorescence, photosynthetically active radiation (PAR), and oxygen saturation (Table 1). Among these parameters, temperature, salinity, and turbidity exhibited seasonal oscillations. Specifically, temperature ranged from 17 to 18.7 °C during winter to 29.4–29.8 °C in summer, showcasing a substantial variance of at least 12.4 °C and 11.1 °C in 2020 and 2021, respectively. Salinity levels peaked at 39.4–39.5 PSU in autumn, nearing the conclusion of the dry season, and descended to a low of 38.5–39 PSU in winter, following periods of precipitation (Fig. S1). Turbidity levels were notably higher during winter samplings (FTU 2.5–2.7) than all other seasons (FTU 0.4–1.1), attributed to frequent winter storms. The day length during microplastic collections varied from 10.34 to 13.58 h in the winter and summer, respectively. All other parameters (fluorescence, photosynthetically active radiation (PAR), and oxygen saturation) demonstrated non-cyclic variation across the sampling intervals, indicating reliance on short-term events rather than seasonal variables.

The microplastic samples (0.3–5 mm in size) of different shapes and colors presented different surface topography (Fig. 1C–K). Across seasons, the composition of microplastic shapes and colors was similar. As depicted in Fig. S2, the majority (53.2–66.3%) of the plastic debris that was sampled throughout the year were films, followed by filaments (24-32.5%) and fragments (5-18.6%). A small percentage (max 5%) were pellets and beads. Color-wise, transparent microplastics were prevalent (51-57.1%), followed by white items (13.4–19.5%), while the remaining portion encompassed microplastics of assorted colors. The microplastic surfaces were covered with patches of biofilm and dominated by unicellular organisms including bacteria (Fig. 1D,E) and diatoms (Fig. 1F–K).

### The biodiversity of microplastic biota

The microbiome analysis of the collected microplastics revealed various prokaryotic and eukaryotic organisms. Community biodiversity parameters, including richness (Chao1 index) and diversity (Shannon’s index) of the 16 S and 18 S metabarcoding datasets, were compared based on sample type (plastic vs. seawater), collection year (2020 vs. 2021) and season (separately for plastic and seawater) (Fig. S3). Microplastic samples showed higher prokaryotic species richness overall than seawater samples (Fig. S3A). In contrast, the 18 S eukaryotic dataset showed an opposite trend with higher eukaryotic richness and diversity in the seawater samples (Fig. S3C, D). No significant differences in biodiversity were observed between 2020 and 2021 in any of the alpha diversity analyses (Fig. S3E-H). Biodiversity seasonal variance was observed in two cases: in the microplastic 16 S dataset, a higher biodiversity was noted in autumn as compared to summer (Fig. S3J), and in the seawater dataset, greater biodiversity was recorded in winter as compared to spring (Fig. S3N). No significant seasonal variation was observed in the 18 S dataset.

**Fig. 2:**
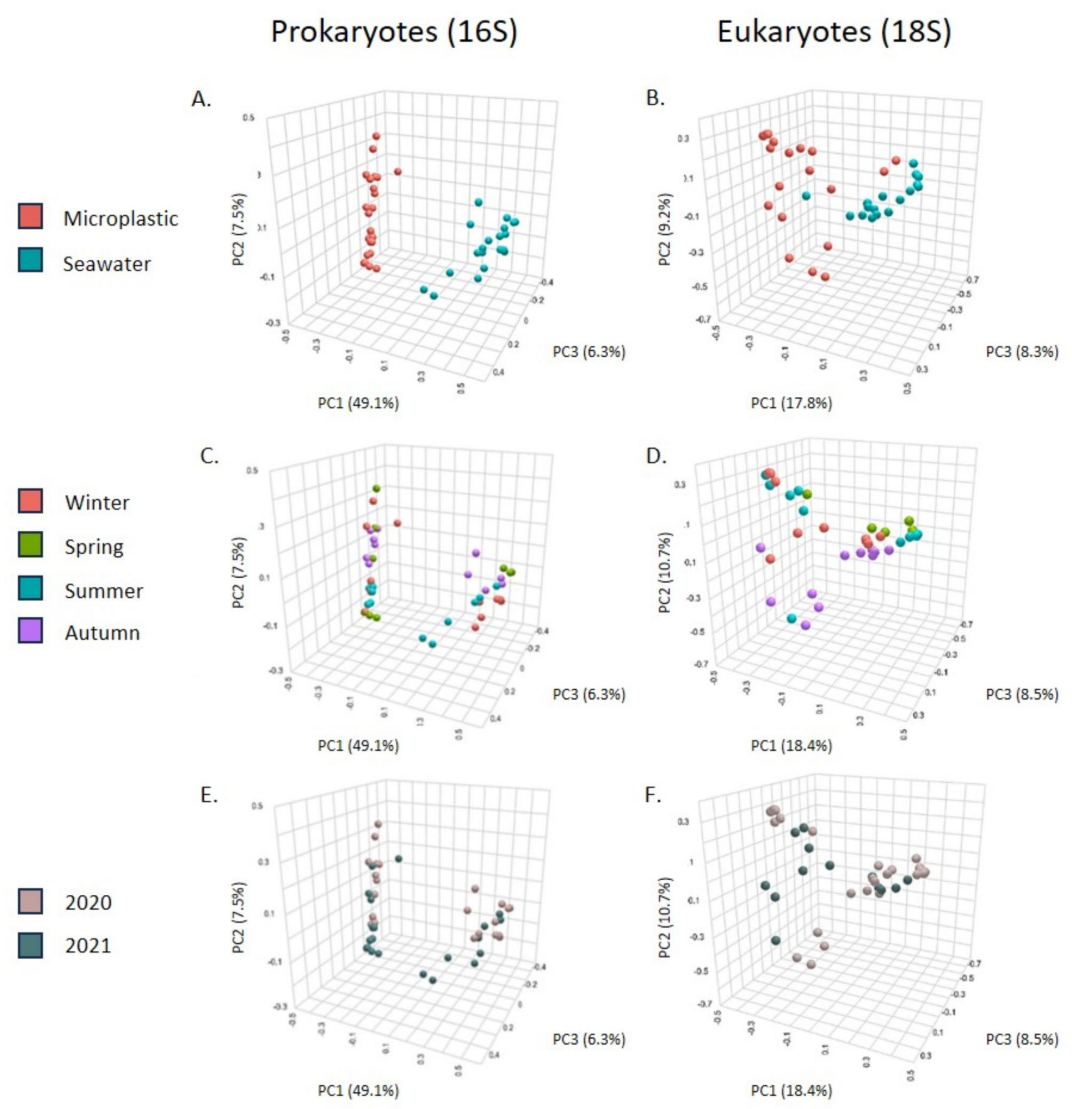
Prokaryotic and eukaryotic biodiversity across samples (beta diversity). On the Left side – Prokaryotes (16 S barcode). On the Right side – Eukaryotes (18 S barcode). (**A**,**B**) Colored by sample type, (**C**,**D**) colored by season, (**E**,**F**) colored by year. Three dimensions represent the three PCA vectors that explain the highest portion of the variance. The figure was created using MicrobiomeAnalyst 2.0 web platform (https://www.microbiomeanalyst.ca/MicrobiomeAnalyst/upload/OtuUploadView.xhtml).

PCA analysis assessed the dissimilarities in the community composition across samples (beta diversity) (Fig. 2). In 16 S and 18 S metabarcoding analyses, seawater samples were distinctly separated from the microplastic samples along component 1 (Fig. 2A,B). However, the distance between the plastic and water samples in the PCA results was greater for the 16 S communities (even more pronounced given that the 16 S PCA component 1 explains 49.1% of the 16 S variance vs. only 17.8% in the 18 S analysis). Seasonal clustering of the samples was also evident to a lesser degree in both the 16 S and 18 S datasets (Fig. 2C,D). Finally, no significant variation was detected between 2020 and 2021 in the analyses considering all samples (see Fig. 2E,F).

### The prokaryotic composition of the microplastic and planktonic microbiome

**Fig. 3:**
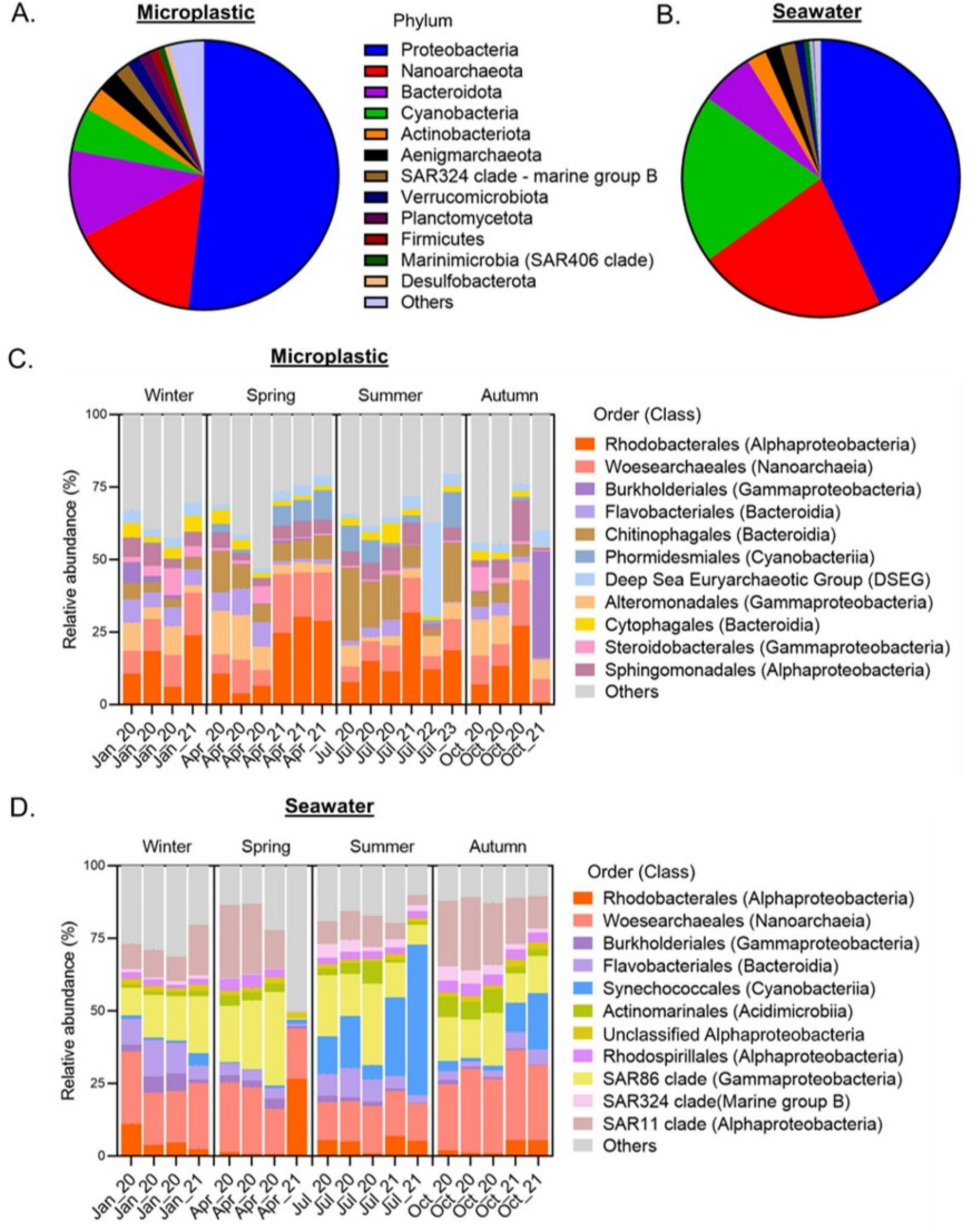
Taxonomic composition of the microplastic prokaryotic microbiota (16S barcode). (**A**) Taxonomic composition of all microplastic samples combined - phylum level. (**B**) Taxonomic composition of all seawater samples combined - phylum level. (**C**) Taxonomic composition of the microplastic microbiome by season – top orders. (**D**) Taxonomic composition of the seawater microbiome by season – top orders. Samples with less than 10k reads were excluded; all other samples were rarified to 10k reads (rarefication curves are shown in Fig. S4).

To depict the overall differences in the prokaryotic taxonomic composition between the microplastic and the seawater samples, we focused on the top 12 most abundant phyla, across all samples and seasons. The analysis revealed differences in the abundance proportions of the dominant phyla between the two environments (Fig. 3A,B). Proteobacteria dominated microplastic and seawater, albeit more prominently on microplastic (51.9% vs. 43% in seawater). Conversely, the phylum Nanoarchaeota was more prevalent in seawater than in microplastic (22% vs. 15.6%, respectively). Bacteroidota emerged as the third most abundant phylum of microplastic (10.6%), whereas it ranked fourth in seawater (6.3%). Interestingly, Cyanobacteria constituted the third most abundant phylum in the planktonic samples, occupying 19.8% of the 16 S barcodes, compared to a mere 5.2% on microplastic. Actinobacteriota, Aenigmarchaeota, SAR324 clade (marine group B), and Verrucomicrobiota appeared in similar proportions in the microplastic and the seawater samples (2.8% vs. 2.3%, 2.6% vs. 1.8%, 1.7% vs. 1.8%, vs. 1.6% vs. 1.1%, respectively). On the other hand, Planctomycetota, Firmicutes, Marinimicrobia, and Desulfobacterota phyla exhibited higher abundance on microplastic (1.4%, 1%, 0.8%, and 0.6%, respectively) as compared to seawater (0.4%, 0.1%, 0.6%, and 0.1%, respectively).

The taxonomic profiles of prokaryotic communities in the microplastic and the seawater samples were further examined to detect seasonal variations specifically on the order level (Fig. 3C,D). Among the top 10 most abundant prokaryote orders, only four orders (Rhodobacterales, Woesearchaeales, Burkholderiales and Flavobacteriales) were shared between microplastic and seawater samples, while others were unique to either plastic or seawater environments. In the microplastic prokaryotic microbiome, the Chitinophagales order exhibited higher relative abundance during spring and summer (8.2% and 14.4% on average) than in autumn and winter (4.1% and 4%). A similar trend was observed for the Cyanobacteria of the order Phormidesiales, with spring and summer showing a higher mean proportion (4.7% and 5.6%, respectively) than winter and autumn (0.4% and 0.2%). In the seawater 16 S microbiome, another order of Cyanobacteria, Synechococcales, was more prevalent during summer and autumn (23% and 7.2% on average) and nearly absent in winter and spring (1.7% and 0.7%). A similar pattern, although less significant, was noted in the seawater dataset for the SAR324 clade, with higher mean relative abundance during summer and autumn (3% and 3.2%, respectively) than in winter and spring (1% and 0.2%, respectively).

### The eukaryotic composition of the microplastic and planktonic microbiome

**Fig. 4:**
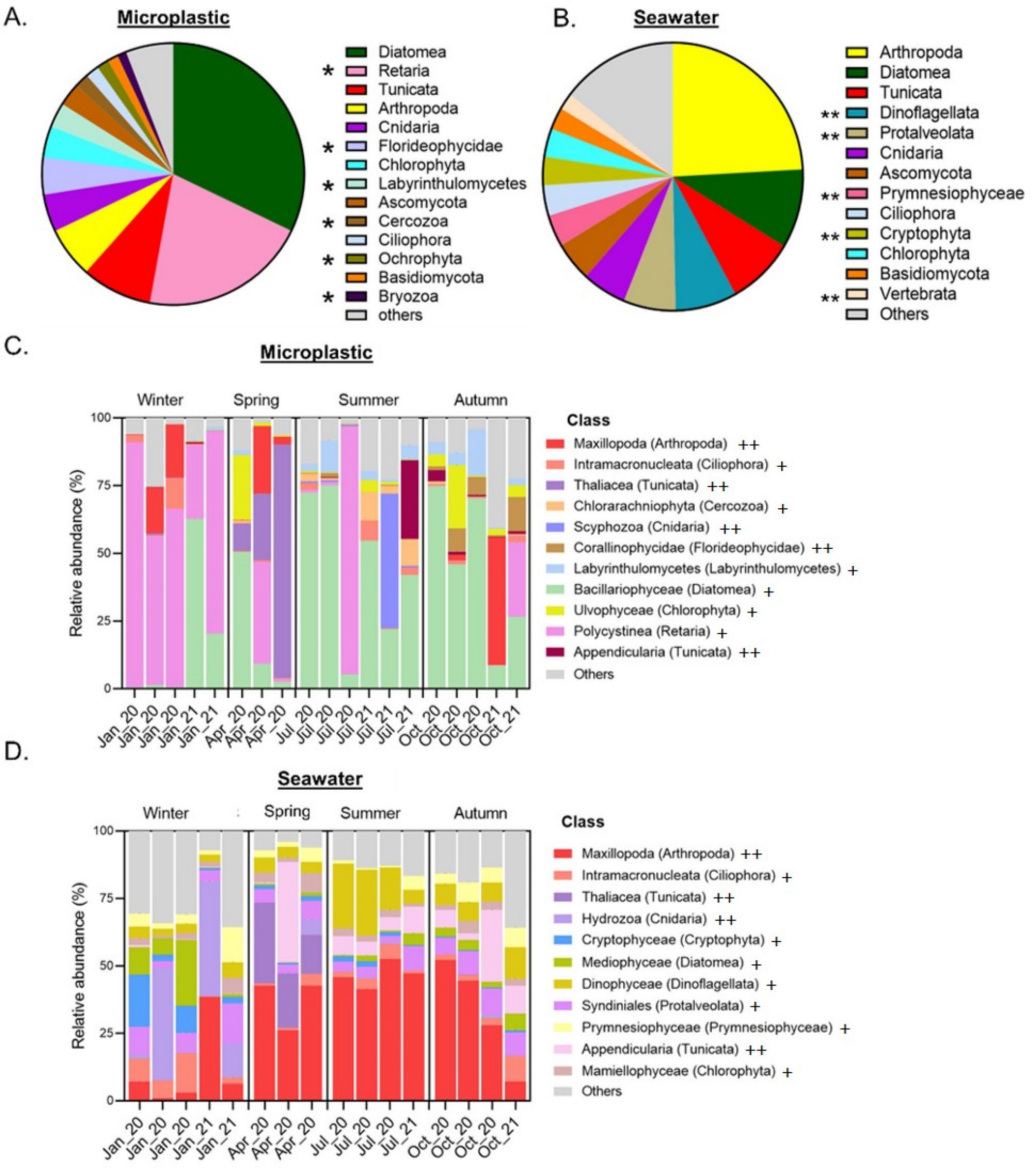
Taxonomic composition of the microplastic eukaryotic biota (18S barcode). (**A**) Taxonomic composition of all microplastic samples combined - phylum level. (**B**) Taxonomic composition of all seawater samples combined - phylum level. *Phyla that were enriched on the microplastic samples and are not shown for the seawater samples, **Phyla that were enriched in the seawater samples and are not shown for the microplastic samples. (**C**) Taxonomic composition of the microplastic biome by season – top 11 classes. (**D**) Taxonomic composition of the seawater biome by season – top 11 classes. Unicellular organisms are marked with ‘+’. Multicellular organisms are marked with ‘++’. Samples with less than 10k reads were excluded; all other samples were rarified to 10k reads (rarefication curves are shown in Fig. S5).

The overall 18 S-based taxonomic composition of the microplastic biome, across all samples and seasons, was compared with that of seawater. It was found that the top abundant phyla differed significantly between the two environments, with several phyla that were unique to either microplastic or seawater (Fig. 4A). The most prevalent phylum on the microplastic was Diatomea, occupying 32.15% of the 18 S barcode matches compared to only 9.55% in the surrounding seawater. Seawater samples had large proportions of Arthropod matches (24.14%), whereas this phylum of multicellular organisms appeared only 4th in the microplastic samples (6.26%). The second most abundant phylum in the microplastic samples was Retaria (20.70%). In contrast, this phylum was below detection in seawater samples. Other top abundant phyla that were unique to microplastic samples included Florideophycidae (4.57%), Labyrinthulomycetes (3.06%), Cercozoa (1.77%), Ochrophyta (1.51%) and Bryozoa (1.13%). Top abundant phyla that were uniquely identified in seawater included Dinoflagellata (7.64%), Protalveolata (6.49%), Prymnesiophyceae (3.86%), Cryptophyta (3.44%) and Vertebrata (2%). In addition, the fungal phyla Ascomycota and Basidiomaycota were relatively more abundant in seawater, comprising 4.77% and 2.67% of the total reads, as compared to 2.81% and 1.30% relative abundance in the microplastic samples.

The taxonomic profiles of the eukaryotic communities in both microplastic and seawater samples were further examined to detect seasonal variations, specifically on the class level (Fig. 4C and D). Bacillariophyceae (diatoms) dominated the microplastic samples of the summer and autumn (both had 45.2% on average of all 18 S reads), while class Polycystinea (Radiolarians) dominated the winter microplastic samples (mean = 62.8%). Tunicates of the Thaliacea class occupied a major part of the spring microplastic and seawater samples (40.2% and 21.6%) and were below threshold abundance in the other seasons. On the other hand, the tunicates of the class Appendicularia were present in the seawater samples during spring, summer and winter (12.6%, 6.6% and 18.5% of the reads) but were almost exclusively restricted to the summer season in the microplastic samples (4.9%). Other classes in the microplastic samples with seasonal appearance included Ulvophyceae (Chlorophyta), Labyrinthulomycetes and Corallinophycidae (Cerozoa). The seawater samples contained high percentages of Maxillopoda (Arthropoda) reads, specifically during the spring and summer (means = 37.2% and 47.8%). Classes Cryptophyceae (Cryptophyta) and Hydrozoa (Cnidaria) appeared mostly in the winter (7% and 19.7%) in the seawater samples but were absent from the microplastic sample analysis.

### Diatoms within the plastisphere exhibit a distinctive taxonomic signature

As mentioned earlier, diatoms were significantly enriched in the microplastic samples, reaching an average of ~ 32% of total 18 S reads as compared to an average of ~ 9% in seawater samples across all time points. In October 2020, diatom 18 S reads within the plastisphere reached a striking ~ 75%. We assume that the proportions of the diatoms within the microplastic eukaryotic community were underestimated in our 18 S metabarcoding analysis because they are unicellular organisms, therefore producing significantly lower copies of 18 S reads per genotype compared to multicellular eukaryotes. SEM imaging of the microplastic samples confirmed significant colonization of diverse diatoms, dominated by a variety of pennate genera occupying pits (Fig. 1F) and scratches (Fig. 1G), attached to a biofilm layer (Fig. 1H) or directly to the plastic surface (Fig. 1I–K).

**Fig. 5:**
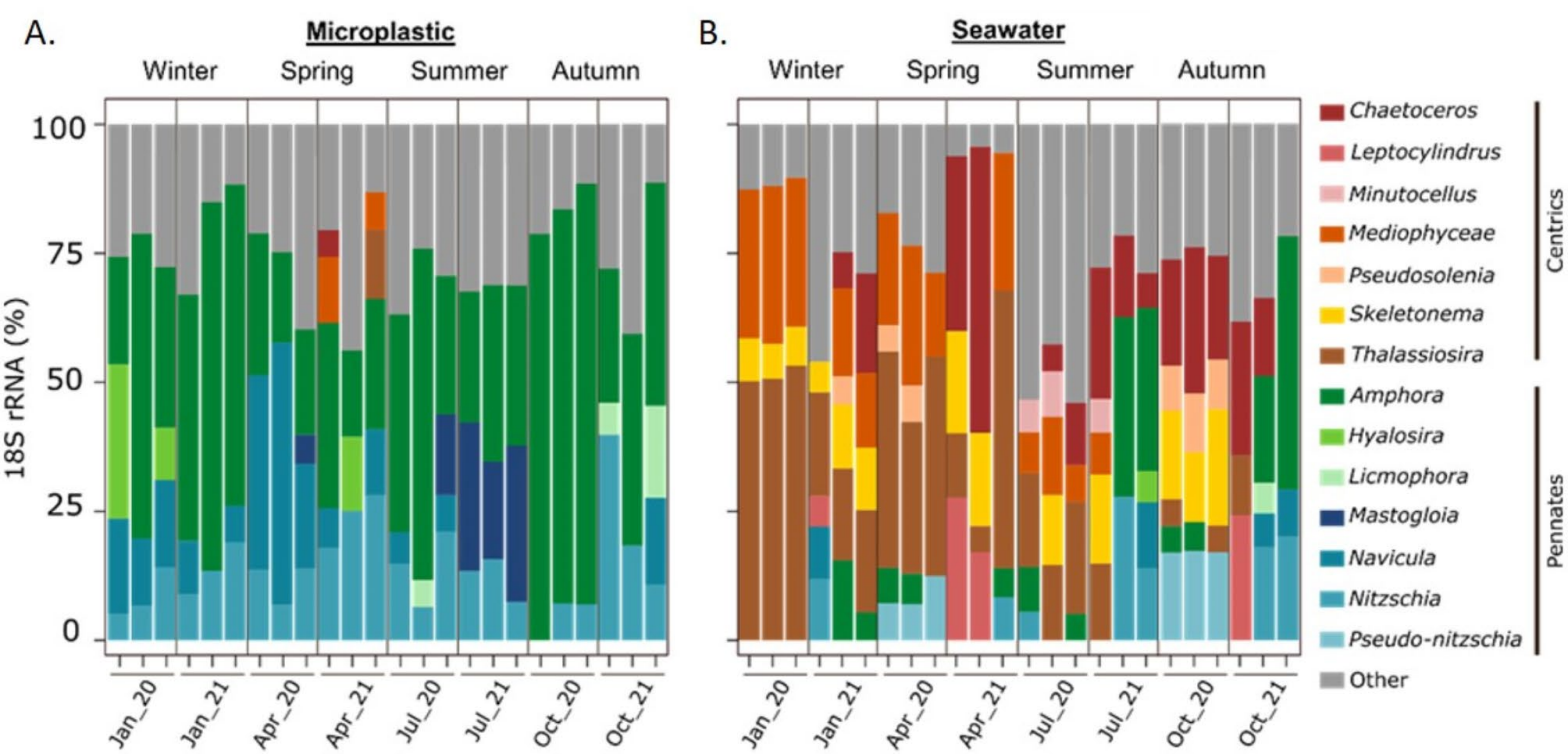
Diatom genera in the plastisphere. Relative 18 S abundance (%) of the top diatom genera across microplastic (**A**) and seawater (**B**) samples. Genera that were present in < 5% relative abundance within a sample or present in < 3 samples were defined as ‘Other’.

The composition of diatom genera in microplastic samples markedly contrasted with that of the surrounding seawater. While the microplastic contained almost exclusively raphid pennate diatoms (> 98%), the water samples were dominated by centric diatom genera (Fig. 5, Fig. S6). Among the diatoms adhering to plastic, *Amphora* was consistently the most abundant genus, ranging from 17 to 82% of diatom OTUs (mean = 41%), with the highest relative abundance in October 2020 and January 2021, followed by *Navicula* (6–51%, mean = 17%), which dominated the April 2020 samples, and *Nitzschia* (5–40%, mean = 15%). *Mastogloia* was predominantly observed in microplastic samples collected in July, ranging from 15 to 30% of the diatom OTUs (Fig. 5A). However, these genera represented a relatively small fraction of the total diatom reads in seawater samples, together making up less than ~ 25% of the 18 S diatom reads in the majority of samples. Notable exceptions are October and July 2021 in which these genera together comprised > 50% of the total reads in two out of three replicate samples (Fig. 5). In contrast with the microplastics diatom community composition, notable diatom genera within the seawater samples included *Thalassiosira*, which comprised 6.1–78% of the diatom reads (mean = 32%), peaking during winter and spring (January and April; Fig. 5B). In addition, both *Skeletonema* (mean = 13%) and *Chaetoceros* (21%) represented a large fraction of the seawater diatom community but did not exhibit significant seasonal variation (Fig. 5B).

### Seasonal variations in species abundance

**Fig. 6:**
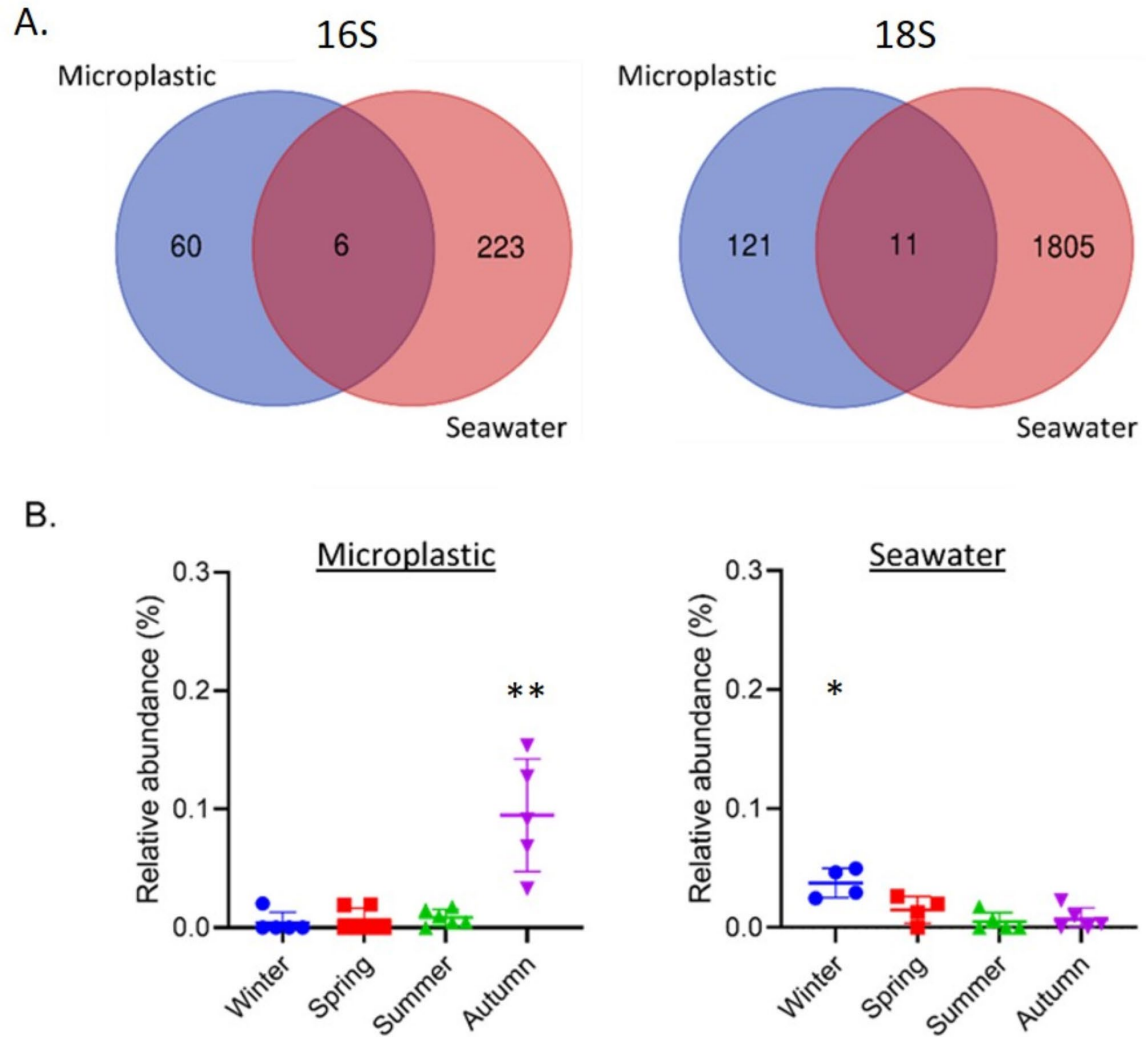
OTUs with significant seasonal abundance variations. (**A**) Venn diagram representation of seasonally significant OTUs determined by Maaslin2 analysis (q-value < 0.05) in the microplastic and the seawater samples, for 16 S/18S barcodes (**B**) Seasonal abundance of a specific OTU that was mapped to *Escherichia-Shigella*. One-way ANOVA, *p-value < 0.05 **p value < 0.01.

To detect species exhibiting significant seasonal fluctuations, we conducted a Maaslin2 analysis based on OTU-level relative abundances as described in the method section. Compared to seawater communities, members of the microplastic community exhibited significantly reduced sensitivity to season, The seawater samples included 14% seasonally variable 16 S OTUs (229 out of 1634) and 10.9% seasonally variable 18 S OTUs (1816 out of 16625) compared with only 1.95% (66 out of 2138) and 3% (132 out of 6748) in the equivalent microplastic sample datasets. Most (60/66) of the seasonally variable microplastic OTUs were unique to microplastic (Fig. 6A). Interestingly, some OTUs exhibited opposing seasonal patterns between the microplastic and seawater samples. For example, *Escherichia-Shigella* exhibited higher relative abundance in microplastic samples during autumn, while it exhibited higher relative abundance in seawater samples during winter (Fig. 6B).

## Discussion

The ocean surface plastisphere community is known to be distinct from its surrounding environment in both its taxonomy and metabolic activity^10^. In this two-year study, we characterized the seasonal differences in the community composition of plastisphere and seawater environments using long 16 S and 18 S rRNA taxonomic barcodes to identify prokaryote and eukaryote taxa.

The Mediterranean Sea, specifically its eastern section, is heavily contaminated with floating microplastic debris^24,25,31^. This debris is constantly exposed to sunlight, supporting colonization by primary producers and the development of a rich microbial community. While the EMS is classified as ultra-oligotrophic^32^, our findings indicate that the bacterial microbiome in the thin microplastic biofilm cover is richer than that in the surrounding seawater, suggesting that the microplastic microbiome may be able to overcome some of the limitations imposed on surrounding planktonic microbial communities. For example, it has been suggested that the plastisphere community overcomes nutrient limitation through continuous internal recycling of essential nutrients, such as nitrogen, iron, and phosphorus^10^. This ‘closed loop’ would essentially create a unique microenvironment within the plastisphere niche that is distinct from the nutrient-poor surrounding seawater^33,34^.

In agreement with previous studies (e.g. Refs.^12,19,23,35,36^), our 16 S metabarcoding results showed an overall higher prevalence of bacteria from the Proteobacteria phylum, mainly of the Alphaproteobacteria and Gammaproteobacteria classes, and the Bacteroidota phylum compared to the surrounding seawater. Interestingly, we identified representation of archaea within the microplastic samples. The archaeal phylum Nanoarchaeota was the second most dominant prokaryotic phylum in both microplastic and seawater samples and the phylum, Aenigmarchaeota, appeared fifth in plastisphere 16 S read abundance. Recent studies have documented the presence of different archaea phyla in multiple marine plastisphere ecosystems^37–39^. Nevertheless, the role of archaea in in plastisphere communities was scarcely studied and warrants further investigation.

It is well established that, alongside spatial effects, seasonality is a major factor in shaping the plastisphere biome^12,19–23^. The EMS exhibit strong seasonal fluctuations in environmental parameters. Surface seawater temperature along the Israeli coast during this study varied by over 10 °C between summer and winter. Salinity also fluctuates between the wet season (winter) and the dry season (summer). Accordingly, we found significant seasonal signatures in the biome composition of our samples. We note that variations were observed among the replicates of the same season within a given year in several instances. This may be attributed to the slightly different sampling locations and some variability among the collected microplastic content. In spite of these variations, seasonal dynamics were evident at all prokaryotic and eukaryotic taxonomic levels of both microplastic and seawater samples. While the EMS plastisphere biome exhibited seasonal signatures, its seasonal variability was significantly lower than in the surrounding seawater. These results suggest that the microplastic microbiome composition is more stable throughout the year and less sensitive to shifting environmental variables. Similarly, a recent study by Nguyen et al.^40^ showed that the plastisphere microbial communities exhibited higher resilience and stability during storm disturbances compared to the surrounding water. Among the seasonally variable bacteria, *E. coli*, a marker for sewage contamination, was enriched on the microplastic during autumn compared to the surrounding seawater. This finding supports the role of microplastic as a potential vector for the dispersal of pathogenic species, as was suggested in previous studies (e.g. Ref.^41^).

Recent work highlighted that eukaryotic taxa may also thrive on floating plastic debris. In the eastern North Pacific subtropical gyre, plastic surfaces were found to be colonized by a diverse array of coastal species, which survive and reproduce in the open ocean, significantly contributing to the composition of the floating plastisphere community^42^. In contrast to prokaryote taxa distribution, we identified several eukaryotic phyla present almost exclusively in the microplastic or seawater samples. The microplastic-specific top abundant phyla included benthic protists and invertebrates of the phyla Retaria, Florideophycidea, Labyrinthulomycetes, Cercozoa, Ochrophita, and Bryozoa, which were not found in the equivalent seawater top list. The floating microplastic debris provides potentially ideal surfaces for colonizing photoautotrophs. Consistent with prior plastisphere microbiome studies^10,43^, diatoms were one of the most prominent groups of photoautotrophs in our microplastic samples. Diatoms are key players in various biogeochemical cycles in the ocean^44^, contributing ~ 20% of primary production globally^45^. Diatoms also contribute substantially to carbon export due to the mineral ballast of their silica-based cell wall^46^. Most plastic contaminants are made of polymers such as low-density polyethylene, polypropylene, and foamed polystyrene that are lighter than seawater and tend to float at the sea surface. The abundant colonization of diatom cells within the plastisphere raises the possibility that diatom silica may ballast microplastics within the water column, by increasing the overall microplastic weight and shifting it buoyancy from positive to negative, facilitating the removal of these particles from the surface ocean. Following the death and decay of these photosynthetic plastisphere organisms in the non-photic zones, the microplastic particles may regain buoyancy and return to the euphotic zone, where they can be recolonized by primary producers, initiating a new cycle^47,48^. Here, we showed that the plastisphere diatom community is enriched with raphid pennate compared to the surrounding seawater diatom community dominated by centric diatom genera. Raphid pennate diatoms, characterized by the presence of a raphe, or small slit in the silica valve that enables motility along surfaces are typically found in coastal, benthic environments, adapted to life on surfaces as opposed to the pelagic ocean^49,50^. Furthermore, raphid pennate diatoms have been previously observed colonizing artificial surfaces^51^. Our observations suggest that the plastisphere may constitute an additional important microenvironment in the pelagic ocean for diatom colonization. Cyanobacteria, on the other hand, were found in relatively lower relative abundance in the microplastic samples. This is in contrast to other Mediterranean Sea studies (e.g. Refs.^36,52^), which may be attributed to the special environmental conditions in the studied EMS location. The microplastic-bound Cyanobacteria were dominated by the order Phormidesmiales, while high proportions of the strictly planktonic *Synechococcus* Cyanobacteria were recorded in the seawater samples.

In conclusion, our study reveals the unique composition of the EMS microplastic microbiome. We found it to be more stable throughout the year than the surrounding seawater, with significant seasonal influences on the abundance and behavior of certain bacteria. The unique microbial and eukaryotic communities on microplastic debris, such as diatoms and archaea, highlight the complex interactions, selective forces and adaptations within the plastisphere, warranting further investigation into the ecological function and biogeochemical impacts of microplastic microbial communities in diverse marine environments.

## Electronic supplementary material

Below is the link to the electronic supplementary material.

Supplementary Material 1

## Data Availability

All Nanopore MinION filtered reads analyzed in this project were deposited in the NCBI SRA database. The 16 S samples were deposited under Bioproject PRJNA1093090 and PRJNA1093073 (for 2020 and 2021, respectively). The 18 S samples were deposited under Bioproject PRJNA1088808 and PRJNA1091618 (for 2020 and 2021, respectively).

## References

[1] Borrelle, S. B. et al. Predicted growth in plastic waste exceeds efforts to mitigate plastic pollution. *Science ***369**, 1515–1518 (2020). 32943526 10.1126/science.aba3656

[2] Lincoln, S. et al. Marine litter and climate change: inextricably connected threats to the world’s oceans. *Sci. Total Environ. ***837**, 155709 (2022). 35525371 10.1016/j.scitotenv.2022.155709

[3] Frias, J. P., Nash, R. & Microplastics Finding a consensus on the definition. *Mar. Pollut. Bull. ***138**, 145–147 (2019). 30660255 10.1016/j.marpolbul.2018.11.022

[4] Shahul Hamid, F. et al. Worldwide distribution and abundance of microplastic: how dire is the situation? *Waste Manag. Res. ***36**, 873–897 (2018). 30103651 10.1177/0734242X18785730

[5] Chen, B. et al. Global distribution of marine microplastics and potential for biodegradation. *J. Hazard. Mater. ***451**, 131198 (2023). 36921415 10.1016/j.jhazmat.2023.131198

[6] Cózar, A. et al. Plastic debris in the open ocean. *Proc. Natl. Acad. Sci. ***111**, 10239–10244 (2014). 24982135 10.1073/pnas.1314705111 PMC4104848

[7] Hale, R. C., Seeley, M. E., Guardia, L., Mai, M. J. & Zeng, E. L. Y. A global perspective on microplastics. *J. Geophys. Res. Oceans ***125**, e2018JC014719 (2020).

[8] Marsay, K. S. et al. High-resolution screening for marine prokaryotes and eukaryotes with selective preference for polyethylene and polyethylene terephthalate surfaces. *Front. Microbiol. ***13**, 845144 (2022). 35495680 10.3389/fmicb.2022.845144 PMC9042255

[9] Zettler, E. R., Mincer, T. J. & Amaral-Zettler, L. A. Life in the plastisphere: microbial communities on plastic marine debris. *Environ. Sci. Technol. ***47**, 7137–7146 (2013). 23745679 10.1021/es401288x

[10] Amaral-Zettler, L. A., Zettler, E. R. & Mincer, T. J. Ecology of the plastisphere. *Nat. Rev. Microbiol. ***18**, 139–151 (2020). 31937947 10.1038/s41579-019-0308-0

[11] Wang, Z. et al. Plastisphere enrich antibiotic resistance genes and potential pathogenic bacteria in sewage with pharmaceuticals. *Sci. Total Environ. ***768**, 144663 (2021). 33454495 10.1016/j.scitotenv.2020.144663

[12] Marsay, K. S. et al. The geographical and seasonal effects on the composition of marine microplastic and its microbial communities: the case study of Israel and Portugal. *Front. Microbiol. ***14**, 1089926 (2023). 36910177 10.3389/fmicb.2023.1089926 PMC9992426

[13] Lv, S., Li, Y., Zhao, S. & Shao, Z. Biodegradation of typical plastics: from Microbial Diversity to metabolic mechanisms. *Int. J. Mol. Sci. ***25**, 593 (2024). 38203764 10.3390/ijms25010593 PMC10778777

[14] Davidov, K. et al. Identification of plastic-associated species in the Mediterranean Sea using DNA metabarcoding with Nanopore MinION. *Sci. Rep. ***10**, 17533 (2020). 33067509 10.1038/s41598-020-74180-z PMC7568539

[15] Pinto, M., Langer, T. M., Hüffer, T., Hofmann, T. & Herndl, G. J. The composition of bacterial communities associated with plastic biofilms differs between different polymers and stages of biofilm succession. *PloS One ***14**, e0217165 (2019). 31166981 10.1371/journal.pone.0217165 PMC6550384

[16] Wen, B. et al. Community structure and functional diversity of the plastisphere in aquaculture waters: does plastic color matter? *Sci. Total Environ. ***740**, 140082 (2020). 32927571 10.1016/j.scitotenv.2020.140082

[17] Bhagwat, G. et al. Exploring the composition and functions of plastic microbiome using whole-genome sequencing. *Environ. Sci. Technol. ***55**, 4899–4913 (2021). 33686859 10.1021/acs.est.0c07952

[18] Jacquin, J. et al. Microbial ecotoxicology of marine plastic debris: a review on colonization and biodegradation by the plastisphere. *Front. Microbiol. ***10**, 865 (2019). 31073297 10.3389/fmicb.2019.00865 PMC6497127

[19] Oberbeckmann, S., Osborn, A. M. & Duhaime, M. B. Microbes on a bottle: substrate, season and geography influence community composition of microbes colonizing marine plastic debris. *PLoS One ***11**, e0159289 (2016). 27487037 10.1371/journal.pone.0159289 PMC4972250

[20] Sérvulo, T. et al. Plastisphere composition in a subtropical estuary: influence of season, incubation time and polymer type on plastic biofouling. *Environ. Pollut. ***332**, 121873 (2023). 37244532 10.1016/j.envpol.2023.121873

[21] Oberbeckmann, S., Loeder, M. G., Gerdts, G. & Osborn, A. M. Spatial and seasonal variation in diversity and structure of microbial biofilms on marine plastics in northern European waters. *FEMS Microbiol. Ecol. ***90**, 478–492 (2014). 25109340 10.1111/1574-6941.12409

[22] Zhang, B. et al. Spatial and seasonal variations in biofilm formation on microplastics in coastal waters. *Sci. Total Environ. ***770**, 145303 (2021). 33515883 10.1016/j.scitotenv.2021.145303

[23] Dong, X. et al. Seasonal biofilm formation on floating microplastics in coastal waters of intensified marinculture area. *Mar. Pollut. Bull. ***171**, 112914 (2021). 34488149 10.1016/j.marpolbul.2021.112914

[24] Sharma, S., Sharma, V. & Chatterjee, S. Microplastics in the Mediterranean Sea: sources, pollution intensity, sea health, and regulatory policies. *Front. Mar. Sci. ***8**, 634934 (2021).

[25] van der Hal, N., Ariel, A. & Angel, D. L. Exceptionally high abundances of microplastics in the oligotrophic Israeli Mediterranean coastal waters. *Mar. Pollut. Bull. ***116**, 151–155 (2017). 28063700 10.1016/j.marpolbul.2016.12.052

[26] Karamitros, T. & Magiorkinis, G. Multiplexed targeted sequencing for oxford nanopore MinION: a detailed library preparation procedure. *Next Generation Sequencing Methods Protocols*, 43–51 (2018). 10.1007/978-1-4939-7514-3_4 29224067

[27] Bolyen, E. et al. Reproducible, interactive, scalable and extensible microbiome data science using QIIME 2. *Nat. Biotechnol. ***37**, 852–857 (2019). 31341288 10.1038/s41587-019-0209-9 PMC7015180

[28] Quast, C. et al. The SILVA ribosomal RNA gene database project: improved data processing and web-based tools. *Nucleic Acids Res. ***41**, D590–D596 (2012). 23193283 10.1093/nar/gks1219 PMC3531112

[29] Lu, Y. et al. MicrobiomeAnalyst 2.0: comprehensive statistical, functional and integrative analysis of microbiome data. *Nucleic Acids Res. ***51**, W310–W318 (2023). 37166960 10.1093/nar/gkad407 PMC10320150

[30] Mallick, H. et al. Multivariable association discovery in population-scale meta-omics studies. *PLoS Comput. Biol. ***17**, e1009442 (2021). 34784344 10.1371/journal.pcbi.1009442 PMC8714082

[31] Cózar, A. et al. Plastic accumulation in the Mediterranean Sea. *PloS One ***10**, e0121762 (2015). 25831129 10.1371/journal.pone.0121762 PMC4382178

[32] Reich, T. et al. A year in the life of the Eastern Mediterranean: monthly dynamics of phytoplankton and bacterioplankton in an ultra-oligotrophic sea. *Deep Sea Res. Part I ***182**, 103720 (2022).

[33] Mincer, T. J., Zettler, E. R. & Amaral-Zettler, L. A. Biofilms on plastic debris and their influence on marine nutrient cycling, productivity, and hazardous chemical mobility. *Hazard. Chem. Assoc. Plast. Mar. Environ.* 221–233 (2019).

[34] Wright, R. J., Erni-Cassola, G., Zadjelovic, V., Latva, M. & Christie-Oleza, J. A. Marine plastic debris: a new surface for microbial colonization. *Environ. Sci. Technol. ***54**, 11657–11672 (2020). 32886491 10.1021/acs.est.0c02305

[35] A Bryant, J. et al. Diversity and activity of communities inhabiting plastic debris in the North Pacific Gyre. *MSystems ***1**, 101128msystems00024–101128msystems00016 (2016). 10.1128/mSystems.00024-16 PMC5069773 27822538

[36] Amaral-Zettler, L. A. et al. Diversity and predicted inter-and intra-domain interactions in the Mediterranean plastisphere. *Environ. Pollut. ***286**, 117439 (2021). 34438479 10.1016/j.envpol.2021.117439

[37] Agostini, L. et al. Deep-sea plastisphere: long-term colonization by plastic-associated bacterial and archaeal communities in the Southwest Atlantic Ocean. *Sci. Total Environ. ***793**, 148335 (2021). 34174607 10.1016/j.scitotenv.2021.148335

[38] Wang, Q. et al. Colonization characteristics and dynamic transition of archaea communities on polyethylene and polypropylene microplastics in the sediments of mangrove ecosystems. *J. Hazard. Mater. ***471**, 134343 (2024). 38640671 10.1016/j.jhazmat.2024.134343

[39] Vaksmaa, A. et al. Microbial communities on plastic polymers in the Mediterranean Sea. *Front. Microbiol. ***12**, 673553 (2021). 34220756 10.3389/fmicb.2021.673553 PMC8243005

[40] Nguyen, D., Masasa, M., Ovadia, O. & Guttman, L. Ecological insights into the resilience of marine plastisphere throughout a storm disturbance. *Sci. Total Environ. ***858**, 159775 (2023). 36309286 10.1016/j.scitotenv.2022.159775

[41] Junaid, M., Siddiqui, J. A., Sadaf, M., Liu, S. & Wang, J. Enrichment and dissemination of bacterial pathogens by microplastics in the aquatic environment. *Sci. Total Environ. ***830**, 154720 (2022). 35337880 10.1016/j.scitotenv.2022.154720

[42] Haram, L. E. et al. Extent and reproduction of coastal species on plastic debris in the North Pacific Subtropical Gyre. *Nat. Ecol. Evol. ***7**, 687–697 (2023). 37069334 10.1038/s41559-023-01997-y PMC10172146

[43] Reisser, J. et al. Millimeter-sized marine plastics: a new pelagic habitat for microorganisms and invertebrates. *PloS One ***9**, e100289 (2014). 24941218 10.1371/journal.pone.0100289 PMC4062529

[44] Benoiston, A. S. et al. The evolution of diatoms and their biogeochemical functions. *Philos. Trans. R. Soc. B Biol. Sci. ***372**, 20160397 (2017). 10.1098/rstb.2016.0397 PMC5516106 28717023

[45] Rousseaux, C. S. & Gregg, W. W. Interannual variation in phytoplankton primary production at a global scale. *Remote Sens. ***6**, 1–19 (2013).

[46] Tréguer, P. et al. Influence of diatom diversity on the ocean biological carbon pump. *Nat. Geosci. ***11**, 27–37 (2018).

[47] Baumas, C. & Bizic, M. A focus on different types of organic particles and their significance in the open ocean carbon cycle. *Prog. Oceanogr.*, 103233 (2024).

[48] Kooi, M., Nes, E. H., v., Scheffer, M. & Koelmans, A. A. Ups and downs in the ocean: effects of biofouling on vertical transport of microplastics. *Environ. Sci. Technol. ***51**, 7963–7971 (2017). 28613852 10.1021/acs.est.6b04702 PMC6150669

[49] Bondoc-Naumovitz, K. G. & Cohn, S. A. *Diatom Gliding Motil.* 77–109 (2021).

[50] Poulsen, N. & Davutoglu, M. G. & Zackova Suchanova, J. In* The Molecular life of Diatoms*, 367–393 (Springer, 2022).

[51] Molino, P. J. & Wetherbee, R. The biology of biofouling diatoms and their role in the development of microbial slimes. *Biofouling ***24**, 365–379 (2008). 18604655 10.1080/08927010802254583

[52] Delacuvellerie, A., Geron, A., Gobert, S. & Wattiez, R. New insights into the functioning and structure of the PE and PP plastispheres from the Mediterranean Sea. *Environ. Pollut. ***295**, 118678 (2022). 34915097 10.1016/j.envpol.2021.118678

