# Supplementary material for: Community composition and seasonal dynamics of microplastic biota in the Eastern Mediterranean Sea
Source: Sci Rep. 2024 Oct 30;14:26131. doi: 10.1038/s41598-024-73281-3 (PMC11526100; doi:10.1038/s41598-024-73281-3)
Supplement: Supplementary file 1 — Supplementary Material 1 [file 41598_2024_73281_MOESM1_ESM.docx]

Community Composition and Seasonal Dynamics of Microplastic Biota in the Eastern Mediterranean Sea

Keren Davidov^1^, Katherine S. Marsay^1^, Sheli Itzahri^1^, Maxim Rubin-Blum^2^, Paula Sobral^3^, Chana F. Kranzler^4^ & Matan Oren*^1^

1. Department of Molecular Biology, Ariel University, Ariel, Israel.

2. Israel Oceanographic and Limnological Research, National Institute of Oceanography, Tel Shikmona, Haifa, Israel.

3. MARE - Marine and Environmental Sciences Centre & ARNET - Aquatic Research Network Associated Laboratory, NOVA School of Science and Technology, NOVA University of Lisbon, Lisbon, Portugal,

4. The Mina and Everard Goodman Faculty of Life Sciences, Bar-Ilan University, Ramat-Gan, Israel.

# Supplementary material

**Supplementary figure S1.** Sea surface temperature and salinity at the time of sampling.

**
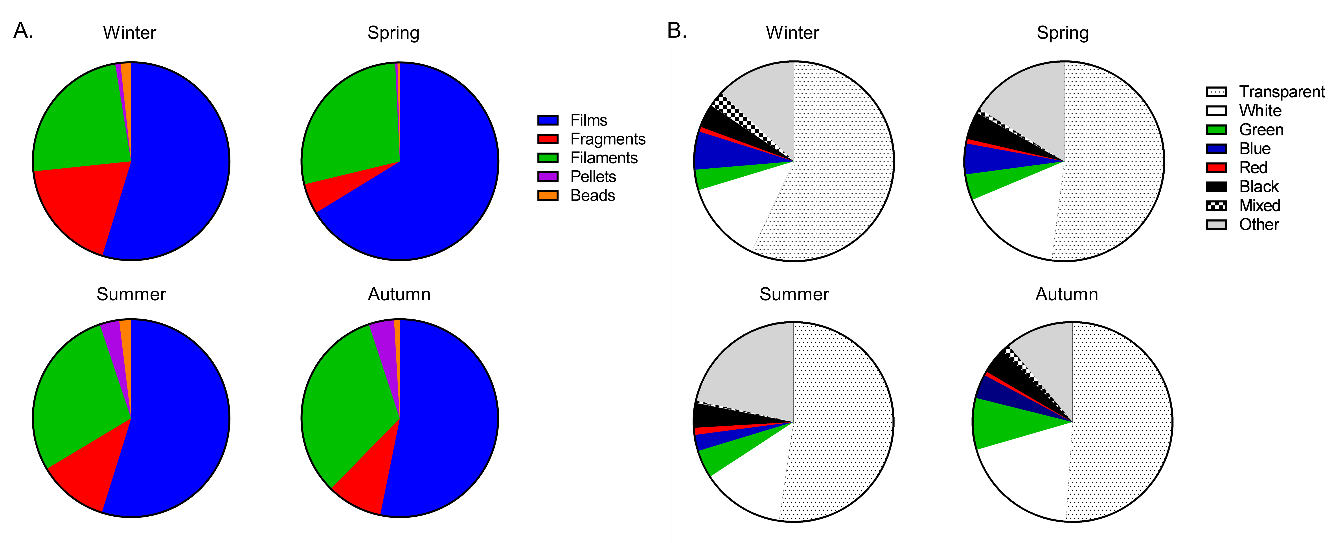
Supplementary figure S2.** Physical characteristics of the microplastic that was sampled during 2021. (A) Abundance of plastic shapes by season, expressed in % relative to total microplastics analyzed (repeats combined) (B)  Plastic abundances by color at every season.


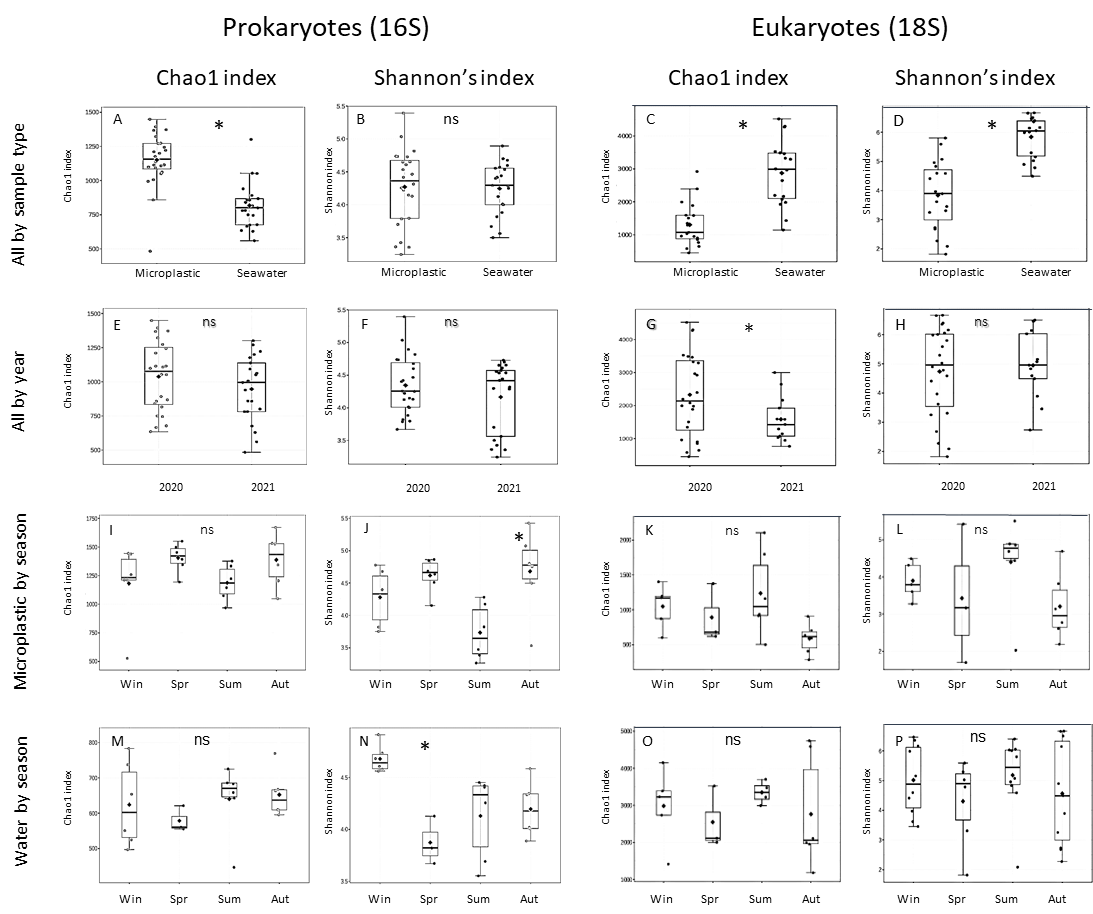


**Supplementary figure S3. Prokaryotic and eukaryotic biodiversity within samples (alpha diversity).** On the left – Prokaryotes (16S barcode). On the right – Eukaryotes (18S barcode). A-D. Biodiversity by sample type (microplastic/seawater), E-H. Biodiversity by sampling year, I-P. Biodiversity by the season of the microplastic samples (I-L) and the seawater samples (M-P). Species richness was assessed by Chao1 index and diversity was assessed by Shannon index.


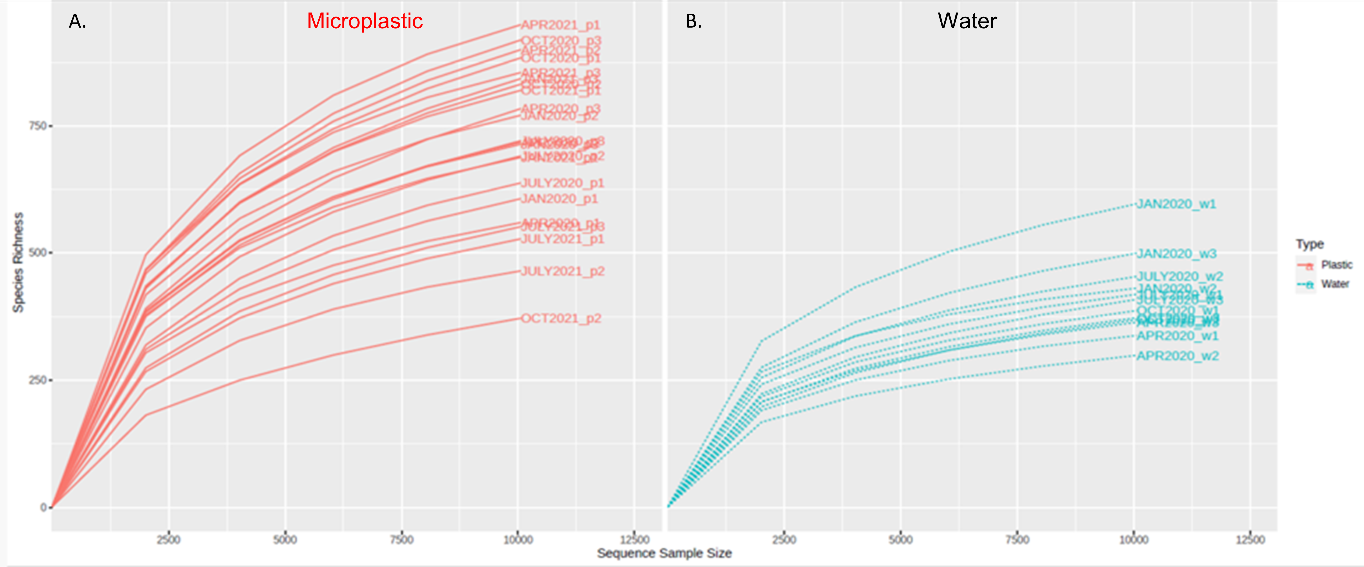
**Supplementary figure S4. Rarefication curves of species richness in the 16S metabarcoding datasets.** The 10,000-read cutoff was chosen based on the sequencing coverage. A. Microplastic samples. B. Water samples.


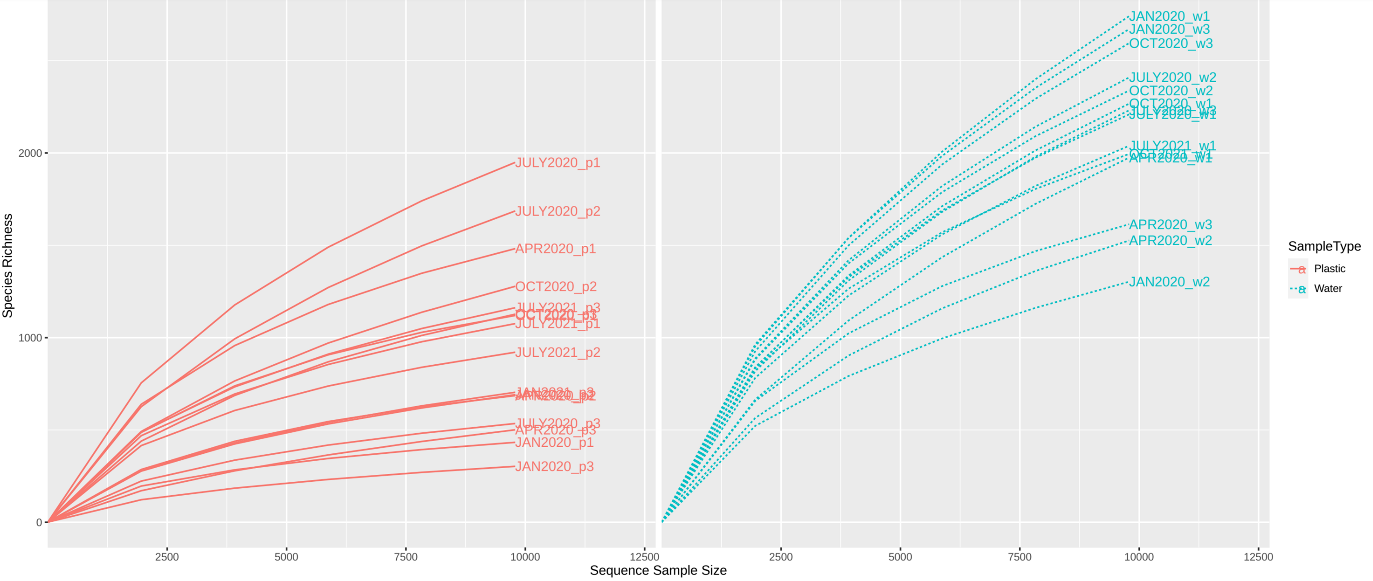


**Supplementary figure S5. Rarefication curves of species richness in the 18S metabarcoding datasets.** The 10,000-read cutoff was chosen based on the sequencing coverage. A. Microplastic samples. B. Water samples.


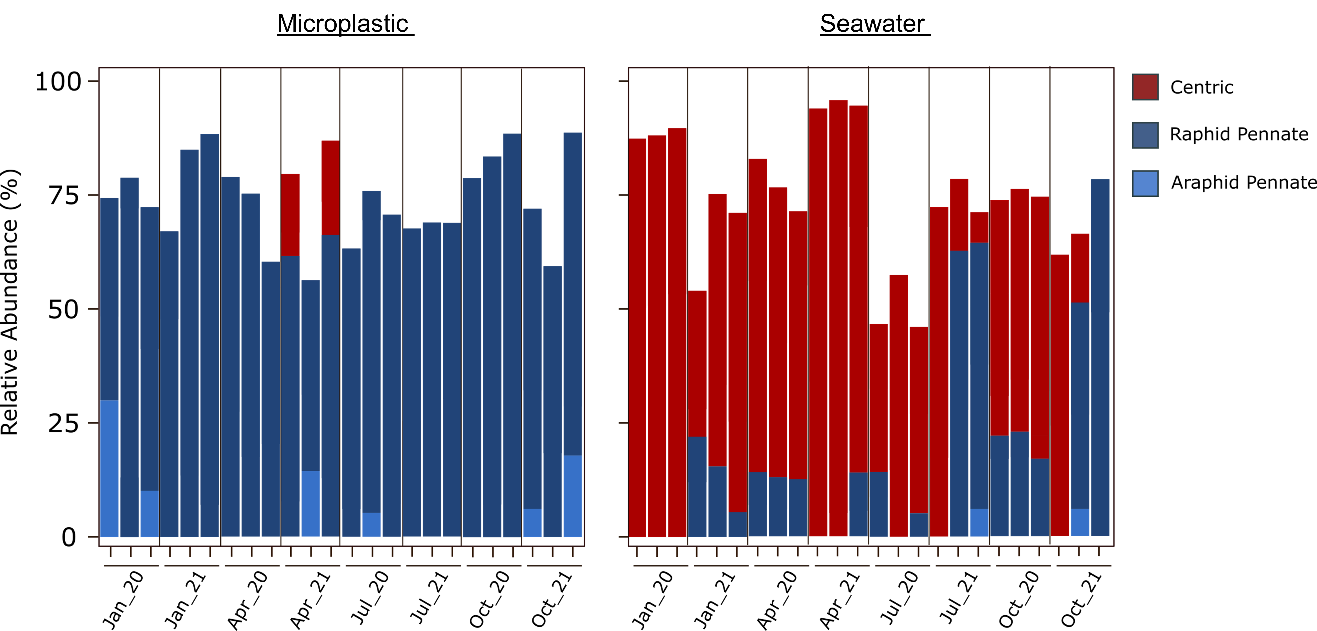


**Supplementary figure S6.** Classification of diatom morphotypes. Relative 18S rRNA abundance of centric (red), raphid pennate (dark blue) and araphid pennate (light blue) diatoms in (a) plastic and (b) whole seawater samples. Graphs show diatom genera with >5% relative abundance and present within at least three samples
